# Systemic Expression of Kaposi Sarcoma Herpesvirus (KSHV) Vflip in Endothelial Cells Leads to a Profound Proinflammatory Phenotype and Myeloid Lineage Remodeling *In Vivo*

**DOI:** 10.1371/journal.ppat.1004581

**Published:** 2015-01-21

**Authors:** Gianna Ballon, Gunkut Akar, Ethel Cesarman

**Affiliations:** Department of Pathology and Laboratory Medicine, Weill Cornell Medical College, New York, New York, United States of America; University of North Carolina at Chapel Hill, UNITED STATES

## Abstract

KSHV is the causative agent of Kaposi sarcoma (KS), a spindle-shaped endothelial cell neoplasm accompanied by an inflammatory infiltrate. To evaluate the role of KSHV vFLIP in the pathogenesis of KS, we constructed mice with inducible expression of vFLIP in endothelial cells. Abnormal cells with endothelial marker expression and fusiform appearance were observed in several tissues reminiscent of the spindle cells found in KS. Serum cytokines displayed a profound perturbation similar to that described in KSHV inflammatory cytokine syndrome (KICS), a recently described clinical condition characterized by elevated IL6 and IL10. An increased myeloid component with suppressive immune phenotype was found, which may contribute to functional changes in the microenvironment and cellular heterogeneity as observed in KS. These mice represent the first in vivo demonstration that vFLIP is capable of inducing vascular abnormalities and changes in host microenvironment with important implications for understanding the pathogenesis and treating KSHV-associated diseases.

## Author Summary

Kaposi’s sarcoma (KS) is the most common cancer in men infected with HIV, and also among the most frequent malignancies in Sub-Equatorial Africa. KS is a tumor of endothelial cell origin that is caused by infection with a gamma-herpesvirus, called KS herpesvirus (KSHV) or human herpesvirus 8 (HHV-8). KSHV vFLIP is a viral oncoprotein expressed during latent infection. We report here the generation and characterization of mice expressing KSHV vFLIP in an inducible manner in endothelial cells. Transgenic mice showed: 1) systemic endothelial abnormalities, with the presence of fusiform cells reminiscent of the spindle cells found in KS, 2) development of a profound perturbation in serum cytokines, reminiscent of the cytokine storm characteristic of KSHV-associated cytokine syndrome (KICS), and 3) remodeling of myeloid differentiation with expansion of myeloid cells displaying a suppressive immunophenotype that potentially favors host immune evasion, angiogenesis and tumor progression. This is the first example of significant changes in myeloid differentiation, vascular abnormalities and cytokine perturbation entirely initiated by ectopic expression of a single viral gene, making this mouse model a useful system to dissect the mechanisms viruses use to manipulate the host microenvironment culminating in sabotage of immunity and development of vascular lesions.

## Introduction

Kaposi sarcoma herpesvirus (KSHV), also called human herpersvirus 8 (HHV-8), one of the most recently discovered human oncoviruses [1], displays tropism for different cell types and a dual oncogenic role, both in lymphomagenesis and vascular oncogenesis. KSHV is specifically associated with Kaposi sarcoma (KS) and two B-cell lymphoproliferative diseases, namely primary effusion lymphoma (PEL) and a large subset of cases of multicentric Castleman’s disease (MCD) [1–3]. KSHV is also associated with KSHV inflammatory cytokine syndrome (KICS), a newly described clinical condition characterized by systemic illness, poor prognosis, elevated KSHV titers, increased levels of viral IL6 and IL10 comparable to those seen in KSHV–MCD but lacking the characteristic lymphadenopathy of KSHV–MCD [4,5], and KSHV-associated hemophagocytic syndrome (VAHS), an extremely rare syndrome reported in immunocompromised patients with MCD and markedly elevated levels of serum human IL6 [6]. KSHV has been found associated also with POEMS syndrome, a rare multisystemic nosological entity characterized by polyneuropathy, organomegaly (particularly cardiomyopathy), endocrinopathy, monoclonal gammopathy and skin lesions [7]; however, a role for KSHV in this disease is controversial, and POEMS may be part of the spectrum of the inflammatory abnormalities seen in MCD, whether KSHV-associated or not.

Similarly to other related herpesviruses, there is dependency on latency for transformation, although this dogma encountered exceptions and has been subjected to debate [8–11]. KSHV genes regulating viral genomic persistence and capable of inducing cellular transformation are transcribed during latency (i.e., LANA, v-cyclin, vFLIP), and the KSHV mode of infection is predominantly latent in KSHV-induced tumors [12]. Experimental data indicate a role for the viral FLICE-inhibitory protein (vFLIP) in KSHV pathogenesis, as it is a latent gene capable of activating NF-κB [13,14], a hallmark cellular pathway constitutively active in PEL and indispensable for the maintenance of lymphoma cell survival [15–17]. FLIP proteins are a group of cellular and viral proteins identified as inhibitors of death-receptor (DR)-induced apoptosis [18,19]. They contain two death effector domains (DED) capable of inhibiting DED-DED interactions between FAS-associated protein with death domain (FADD) and pro-caspase 8 and 10 within the death-inducing signaling complex (DISC) responsible for DR-induced apoptosis [20]. Based on the homology of KSHV vFLIP with cFLIP proteins, it has been thought that vFLIP becomes part of the DISC, preventing the recruitment and processing of procaspase 8 and, thereby, FAS-induced apoptosis [19], although there is little experimental evidence supporting this direct role in apoptosis inhibition. Nonetheless, it is clear is that vFLIP directly binds to IκB kinase (IKK) γ, inducing IKKα/β phosphorylation, IκBα degradation and p100 cleavage, resulting in the activation of both the classical and alternative NF-κB pathways [13,14,21]. Another established function of vFLIP is inhibition of cell death by blocking autophagy [22].

Several groups have developed mice expressing vFLIP in B-cells [23–25]. Among these, our group used a Cre-Lox recombination approach to express vFLIP in all B-cells and specifically in germinal center B-cells, confirming its role in lymphomagenesis and defining the *in vivo* immunological functions of vFLIP as an abrogator of germinal center formation and immunoglobulin (Ig) maturation [23]. Tumors occurring in mice expressing vFLIP in B cells retain major features of PEL, namely B-cell origin, as formally demonstrated by the presence of monoclonal Ig gene rearrangements, and remodeling of BCR with downregulation of B-cell markers, including CD19 and lambda. However, these tumors were also characterized by expression of histiocytic/dendritic cell (DC) antigens, consistent with transdifferentiation from B-cells into the myeloid lineage, without excluding a coexisting paracrine effect on the surrounding myeloid cells [23]. Notably, KS lesions are characterized by the presence of inflammatory cells, including numerous histiocytes [26]. Thus, induction of myeloid cell proliferation by vFLIP could be part of the cellular events and microenvironment alterations that occur during KS pathogenesis.

The role of vFLIP in vascular oncogenesis is suggested by the *in vitro* observations that vFLIP induces spindle cell morphology and expression of inflammatory cytokines in endothelial cells and phosphorylation of STAT1 and STAT2|[27–29]. Both spindling and a proinflammatory microenvironment are key features of KS, defined as a chronic inflammation-associated malignancy due to the presence of spindle-shaped endothelial cells, slit-like neovascular structures, and abnormal vascular spaces with extravasation of red blood cells, as well as variable quantities of infiltrating inflammatory cells and secretion of angiogenic and inflammatory cytokines such as VEGF, PDGF, bFGF, TGFβ, IL1β, IL6 and INFγ [30].

However, the role of vFLIP in the initiation of KSHV-related vascular pathogenesis, if any, is largely unknown. A substantial number of studies have indicated that the cell of origin of KS spindle cells is of endothelial origin as these cells express both blood (*e.g.*, CD34) and lymphatic (*e.g.*, VEGFR3, podoplasmin, LYVE-1, Prox1) endothelial cell markers (BEC, LEC) [31–34] and display a gene signature that falls in between the two cell types, albeit closer to LEC [35]. KSHV can infect both BECs and LECs and is capable of reprogramming their transcriptomes to make BECs more alike LECs and *viceversa* [35–37]. Therefore, to address the role of vFLIP in vascular oncogenesis, we generated mice that express vFLIP under the control of VE-Cadherin promoter, which has been reported to be active in both BECs and LECs [38]. These transgenic (TG) mice showed systemic endothelial alterations with increased spindle-like cells and changes in serum cytokines, reminiscent of certain features of KS and KICS. We also observed remodeling of myeloid differentiation toward cell types known to have implications in host microenvironment, tumor immune evasion, angiogenesis and vascular lesion development.

## Results

### Generation of genetically engineered mice for inducible vFLIP expression in endothelial cells

**Figure 1:**
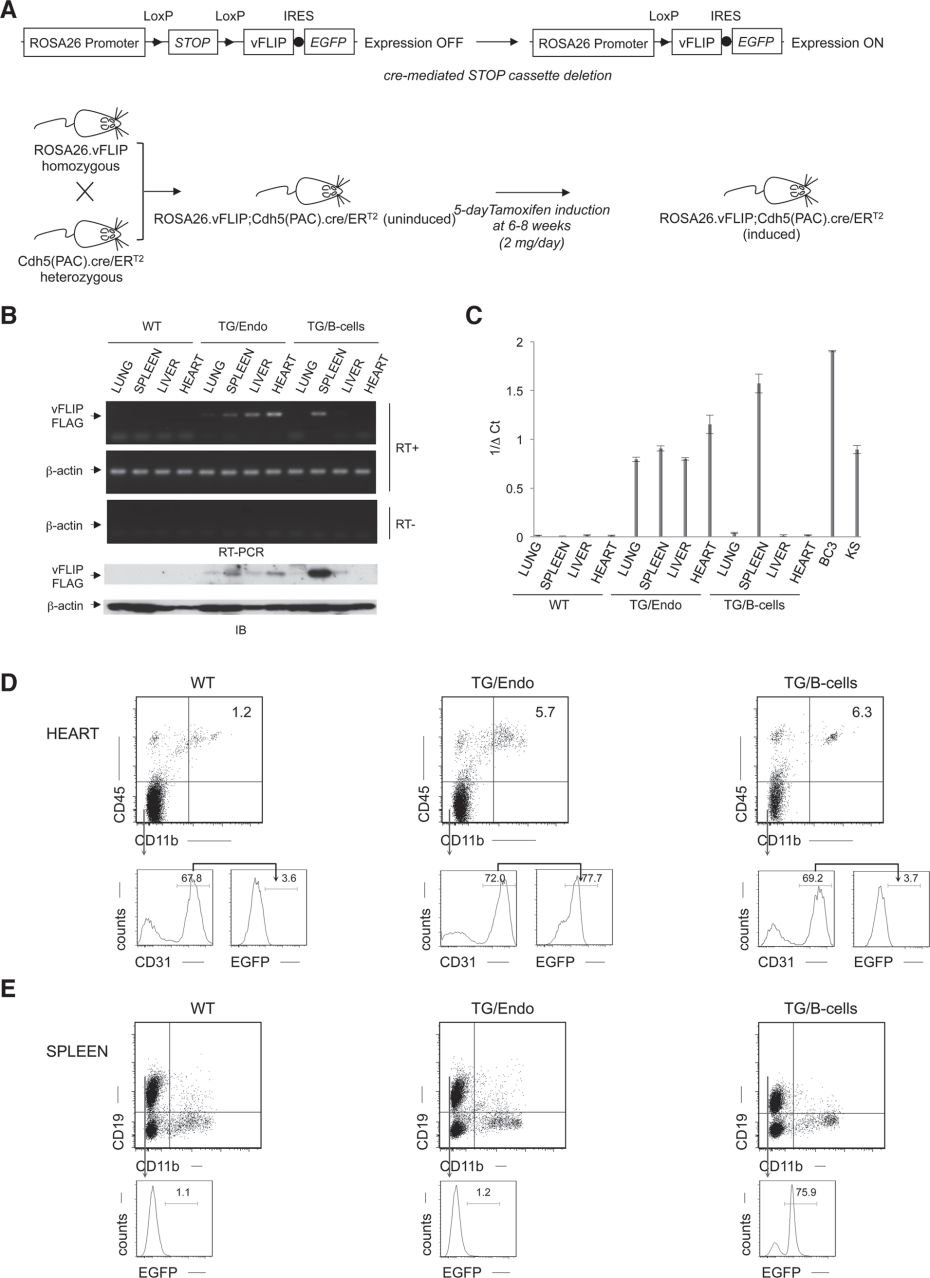
Generation of ROSA26.vFLIP;Cdh5(PAC).creER^T2^ mice. (A) The strategy for inducible recombinant activation of vFLIP expression in endothelial cells *in vivo* is shown with a schematic representation of the ROSA26 locus before (left) and after (right) inducible recombinant activation of vFLIP expression in endothelial cells. ROSA26.vFLIP knock-in mice were bred with Cdh5(PAC).creER^T2^ mice to obtain transgene expression in endothelial cells upon tamoxifen injection. (B) Transgene expression was specifically detected, both by RT-PCR (left panel) and anti-FLAG immunoblotting (right panel), in lung, spleen, liver and heart derived from ROSA26.vFLIP;Cdh5(PAC).creER^T2^. (C) Quantitative real-time RT-PCR for vFLIP expression. Analysis was done in 2–3 month-old mice, about one month after *i.p.* injection of tamoxifen. (D) Flow cytometry showing percentage of cardiac endothelial cells and (E) splenic B-cells expressing EGFP. Data represent one of three experiments with similar results; at least three TG and control animals were analyzed in each experiment. Analysis was done in 2–3 month-old mice, about one month after *i.p.* injection of tamoxifen. TG/Endo are ROSA26.vFLIP;Cdh5(PAC).creER^T2^ mice; TG/B-cells represent ROSA26.vFLIP;CD19.cre mice used as control [23].

We generated mice expressing vFLIP in endothelial cells by using a recombinant inducible system. Previously generated conditional mice for vFLIP (ROSA26.vFLIP knock-in mice) [23] were bred with mice expressing cre recombinase in the form of a fusion protein with the estrogen receptor under the transcriptional control of VE-Cadherin promoter (Cdh5(PAC).creER^T2^ mice) [39], thus resulting in vFLIP expression in endothelial cells upon tamoxifen treatment (Fig. 1A). Before generating ROSA26.vFLIP;Cdh5(PAC).creER^T2^ TG mice, we tried to constitutively express vFLIP in endothelial cells by crossing ROSA26.vFLIP mice with mice expressing cre recombinase under the control of Tie2 promoter. However, embryonic lethality was observed, suggesting that constitutive expression of vFLIP is detrimental for embryogenesis and incompatible with life. Instead, the inducible ROSA26.vFLIP;Cdh5(PAC).creER^T2^ TG mice (carrying both cre and vFLIP) were born at the expected Mendelian frequency and were indistinguishable from their wild-type (WT) littermate controls (carrying only vFLIP) in terms of fertility and developmental features. Expression of vFLIP was evaluated in 2–3 month-old mice, approximately one month after intra-peritoneal (*i.p.*) injection of tamoxifen in both TG and littermate control mice. vFLIP expression was detected at the RNA and protein level in lung, spleen, liver and heart (Fig. 1B).

The level of vFLIP expression was assessed by quantitative real-time RT-PCR in lung, spleen, liver and heart derived from both TG and controls mice, as well as in BC3 PEL cell line and primary KS with lymph node involvement (Fig. 1C). As expected, the highest level of expression was observed in BC3, where all the cells harbor KSHV multiple copies of the viral genome. The splenic fraction derived from B-cell-specific TG mice show also high level of vFLIP expression, comparable with BC3, and this reflects with the high percentage of B-cells in the spleen and the fact that vFLIP expression is controlled by a strong promoter *(i.e.*, CD19). Instead, the endothelial-specific TG mice express lower levels of vFLIP, although comparable with vFLIP expression seen in primary KS. This is consistent with the percentage of endothelial cells in the organs analyzed, which is lower than the percentage of splenic B-cells.

Since antibodies to vFLIP are not adequate for immunohistochemistry or flow cytometry, we monitored transgene expression using antibodies to EGFP, which is expressed in a common transcript with vFLIP due to the insertion of an IRES between the two gene sequences (Fig. 1A). EGFP was detected by immunohistochemistry in cells lining vascular spaces and with the morphologic appearance of endothelial cells in different organs, including intestine (S1A Fig.); these cells were also positive for the endothelial marker CD34. While B-cell-specific TG mice expressed vFLIP in the splenic B-zone as expected, the endothelial-specific TG mice expressed vFLIP in the vascularized interfollicular area (S1B Fig.).

The endothelial identity of transgenic cells and the endothelial specificity of vFLIP expression was confirmed by flow cytometry performed in the heart, where EGFP was expressed in the vast majority (70.8% ± 1.4%), of endothelial cells defined as CD45^−^CD11b^−^CD31^+^ (Fig. 1D, middle panel), but not in splenic B-cells (Fig. 1E, middle panel). Conversely, B-cell specific vFLIP TG mice expressed EGFP in splenic B-cells (Fig. 1E, right panel), but not in endothelial cells (Fig. 1D, right panel). Taken together, these data showed that vFLIP had the expected pattern of expression restricted to endothelial cells.

### vFLIP expression in endothelial cells leads to systemic inflammatory changes and vascular abnormalities

**Figure 2:**
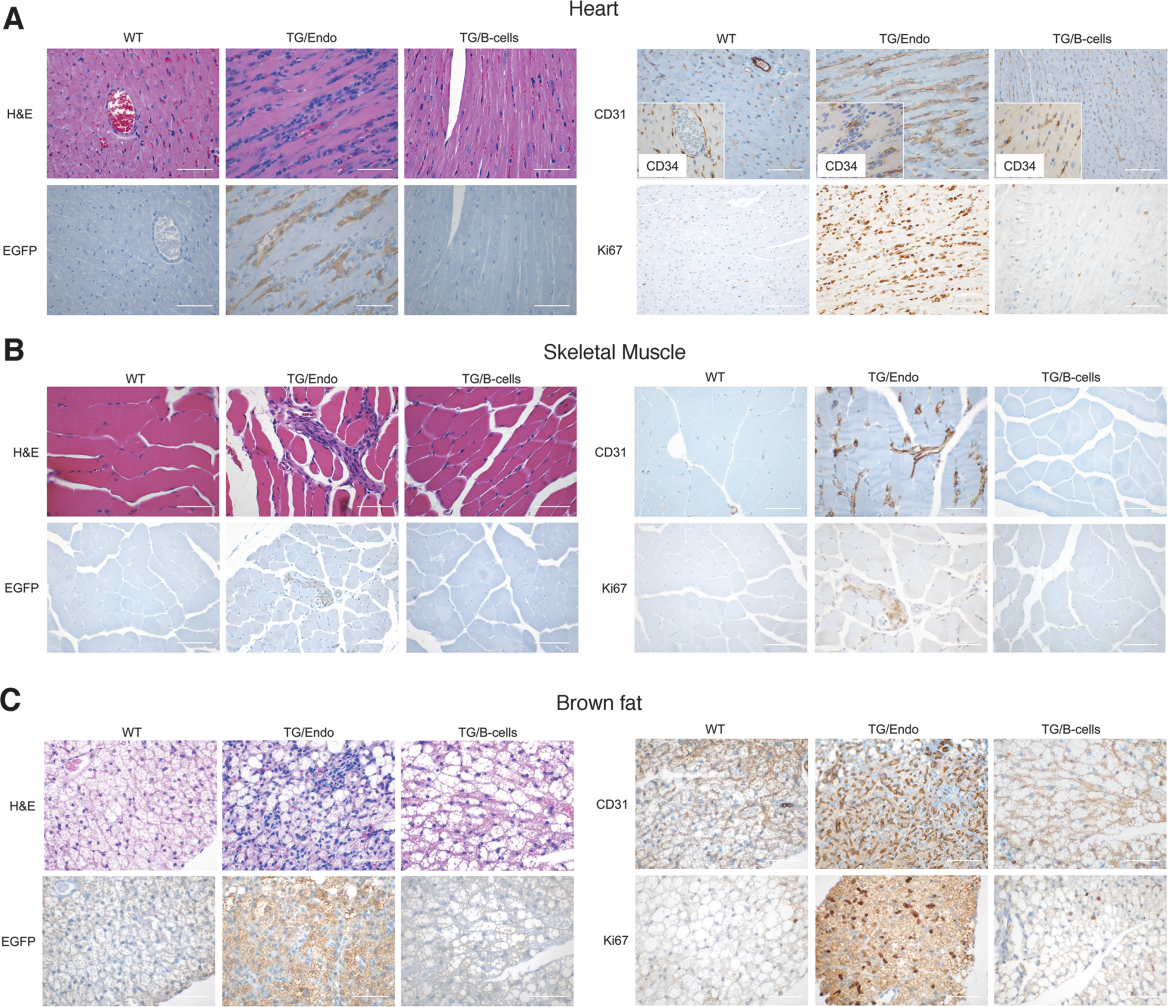
Systemic endothelial abnormalities. (A) A representative cardiac section stained with H&E, EGFP and CD34 and/or CD31, PROX1 and Ki67 shows numerous atypical spindle-like endothelial cells. Similar cells were found in the skeletal muscle (B) and in brown fat (C). Analysis was done in 2–3 month-old mice, about one month after *i.p.* injection of tamoxifen. Scale bar, 200 μm. TG/Endo, ROSA26.vFLIP;Cdh5(PAC).creER^T2^; TG/B-cells, ROSA26.vFLIP;CD19.cre mice used as control.

**Figure 3:**
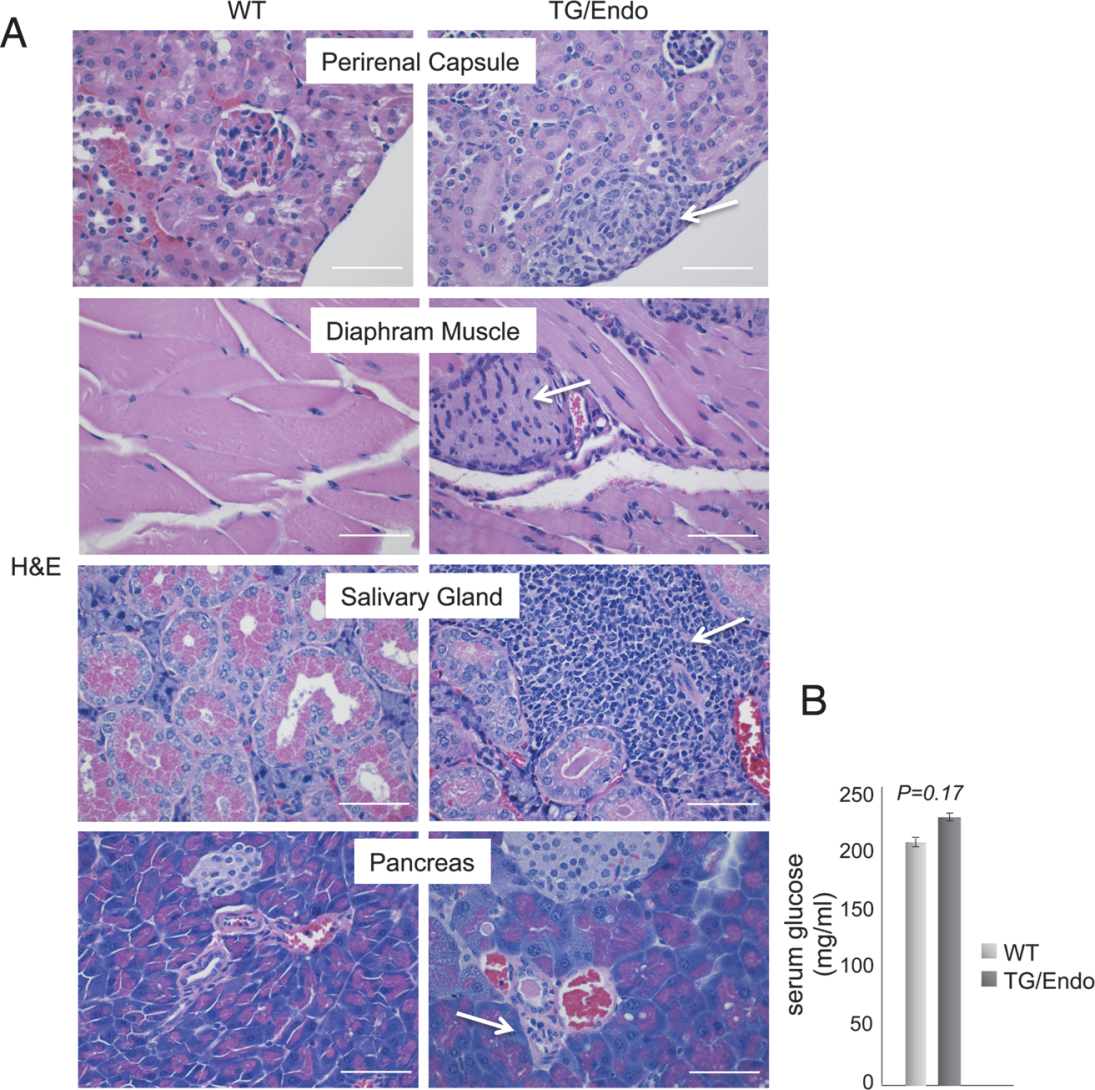
Systemic perineurinal proliferation. (A) Representative section of the perirenal capsule, diaphram muscle, salivary gland, and pancreas stained with H&E shows atypical proliferation of perineurinal endothelial-like cells (arrows). Analysis was done in 2–3 month-old mice, about one month after *i.p.* injection of tamoxifen. Scale bar, 200 μm. (B) Serum glucose levels are shown. Data represent one of three experiments with similar results (error bars, SEM); at least three TG and control animals were analyzed in each experiment.

**Figure 4:**
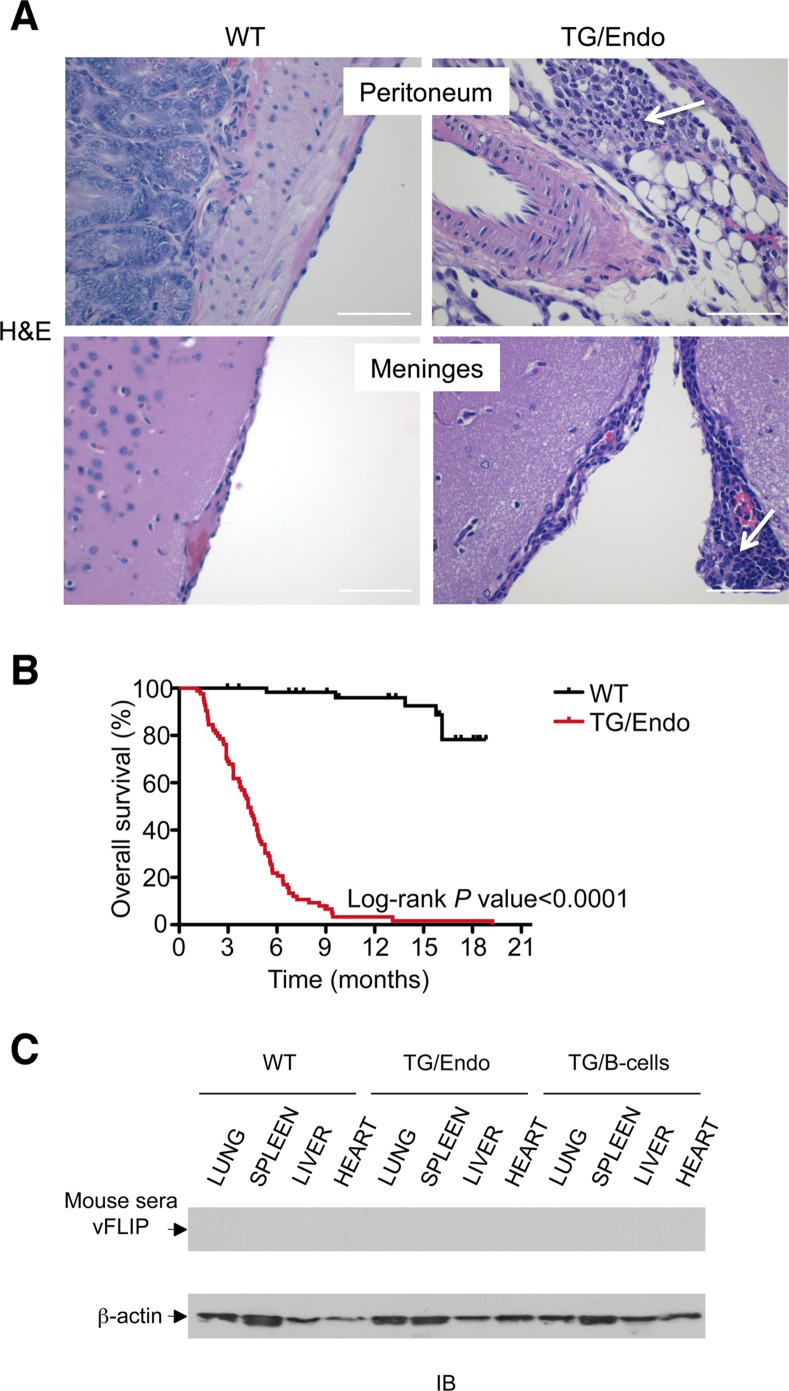
Systemic chronic inflammation. (A) Mixed infiltrates of inflammatory cells including PMN, lymphocytes and plasma cells are shown in peritoneum and meninges (arrows). Scale bar, 200 μm. (B) Statistical analysis of event-free survival by Kaplan-Meier cumulative survival curve and the log-rank test to evaluate statistical significance. More than 100 mice in each group were followed up for up to 20 months. (C) Humoral immune response against vFLIP was tested by western blot with a pool of mouse sera (1:100 dilution) isolated from TG mice. Two independent western blots yielded the same results, and shown here is the one that was displayed also in Fig. 1B.

Virtually all organs and tissues were affected by pathological changes ultimately related to endothelial dysregulation. Numerous elongated cells frequently lining poorly formed vascular spaces was diffusely found throughout several organs, but most notably in the myocardial parenchyma of TG mice. In the heart, these endothelial cells lined the capillaries surrounding individual myofibers, but also proliferated into the parenchyma, expressed vFLIP and many retained endothelial markers (CD34 and/or CD31) and expressed Ki67 (Fig. 2A). Similar findings with the presence of spindle cells, and plump endothelial cells lining vascular spaces, were found in several organs including skeletal muscles (Fig. 2B), brown fat (Fig. 2C) and brain (S2 Fig.). These proliferating endothelial cells do not express the lymphatic marker PROX1, in spite of successful staining of lymphatic endothelial cells in sites where these normally occur including skin, intestines and splenic red pulp (S3A-S3C Fig.). An abnormal perineurial proliferation of endothelial-like cells was found in several tissues in the TG mice, including perirenal capsule, diaphragm muscle, salivary gland, pancreas, but not in the controls (Fig. 3). The abnormalities observed in the pancreas prompted us to check for signs of endocrinopathy (*e.g.*, diabetes); serum glucose levels were slightly increased, but the difference was not significant (Fig. 3). On the abdominal side of diaphragm and in the peripancreatic region, few nerve bundles were also surrounded by hyperplastic perineurial cells, mixed inflammatory cells, lymphocytes and plasma cells and inflammation extended to the adjacent connective tissue. Chronic inflammation, documented with the presence of mixed cell infiltrate of neutrophils, lymphocytes, plasma cells and histiocytes, was found in several tissues, including the peritoneum (Fig. 4A), meninges (Fig. 4A), kidney and skeletal muscle. Both kidneys showed subcapsular areas with numerous spindle cells (Fig. 3), and the perirenal fat was infiltrated by neutrophils, lymphocytes, plasma cells and histiocytes. Extra-medullary hematopoiesis, with both erythroid and myeloid hyperplasia, was present in the spleen and liver. Peripheral blood analysis showed that TG mice have left-shift (*i.e.*, high metamyelocytes and bands with normal neutrophil count), suggesting a demand for neutrophils that exceeded their production and release, a scenario usually seen in case of chronic inflammation at different anatomic sites as observed in our TG mice.

The mice were viable after tamoxifen administration, but starting as early as few weeks after induction they developed the pathological abnormalities here described and by the age of 3–4 months more than 60% of mice had died (Fig. 4B) as result of a systemic illness that comprised myocardial, meningeal, skeletal muscular, peritoneal and perineurial pathological changes. Although i) the pattern of cytokine perturbation indicates the existence of M2-type polarization, which eventually favors immune suppression and tumor immune evasion rather than autoimmunity, [ii) vFLIP does not appear to be a particularly immunogenic protein and iii) KSHV, in general, has developed a wide array of strategies to evade the host immune responses, the mice were not exposed to the transgene during their embryonic development and, thus, they could have theoretically developed immune response toward vFLIP, resulting in a pseudo-autoimmunity that could partially account for the pathological findings and the poor mouse overall survival. Thus, we assessed the presence of a humoral immune response against vFLIP by immunoblotting, but no cross-reactivity was found between a pool of mouse sera isolated from seven TG mice and whole cell lysates derived from lung, spleen, liver and heart of both TG and control mice (Fig. 4C).

### vFLIP induces a cytokine storm reminiscent of KSHV-associated inflammatory cytokine syndrome

**Figure 5:**
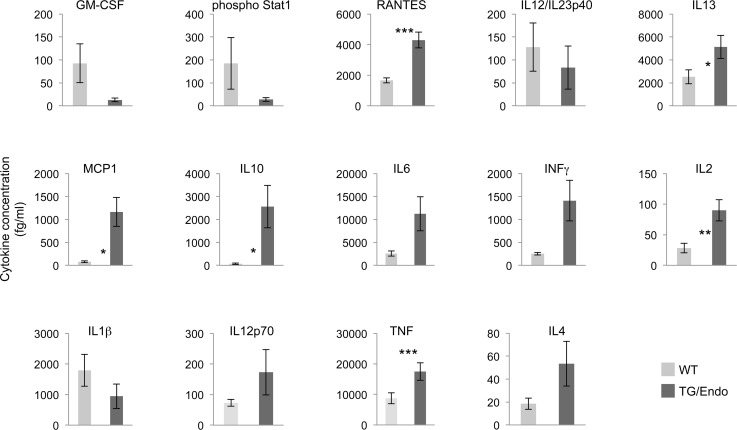
Perturbation in serum cytokines. Fourteen serum cytokines were analyzed in ROSA26.vFLIP;Cdh5(PAC).creER^T2^ mice by using a quantitative flow cytometry-based assay. Analysis was done in 2–3 month-old mice, about one month after *i.p.* injection of tamoxifen. Data are representative of at least three experiments with similar results (error bars, SEM); at least three TG and control animals were analyzed in each experiment. *P*-values derived from two-tailed unpaired Student’s t-test on the means (bars) of WT versus TG mice are shown. *P<0.05, **P<0.01 and ***P<0.005

*In vitro* ectopic expression of vFLIP in either endothelial or B-cells has been shown to confer a myeloid-prone gene expression profile with production of cytokines that have potential tropism for myeloid cells [28,40]. To assess whether *in vivo* expression of vFLIP is capable of exerting similar effects, a panel of fourteen cytokines and growth factors (IL10, IL6, INFγ, IL1β, IL12p70, TNF, IL4, IL2, IL13, GM-CSF, Phospho Stat1, RANTES, IL12/IL23p40, MCP1) was tested in serum samples collected from mice one month after vFLIP induction by tamoxifen (Fig. 5). We used a flow cytometry bead-based assay, which provides quantitative data and is linear within a large range of concentration (from 30 fg/ml to 200000 fg/ml) (S4 Fig.). Compared to control mice, vFLIP TG mice showed increase of IL10, IL6, IL2, IL13, INFγ, TNF, MCP1 and RANTES. These findings are in line with *in vitro* data on gene expression profiling obtained in PEL and endothelial cells that ascribed to vFLIP the ability to activate the expression of several cytokines and growth factors potentially implicated in remodeling of the tumor microenvironment by myeloid cells [28,40]. Noteworthy, the systemic illness with poor prognosis and the profound changes in cytokines profile, particularly with increased IL6 and IL10, are aspects similar to those described for MCD and KICS.

### vFLIP induces remodeling of myeloid differentiation and expansion of CD11b^+^Gr1^+^ cells with suppressor immune phenotype

**Figure 6:**
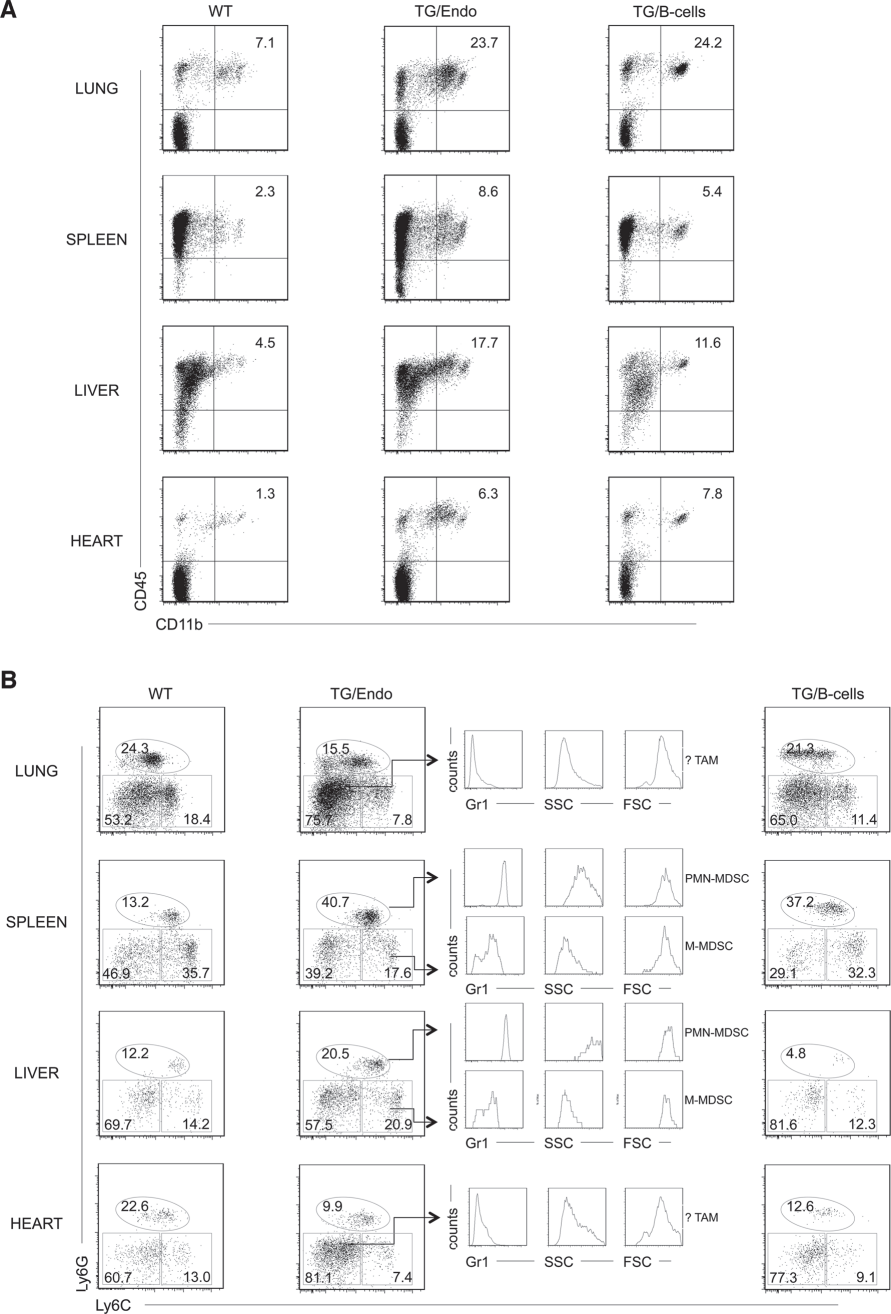
Expansion of myeloid cells with PMN-MDSC immunophenotype. (A) Flow cytometry analysis displayed increase in CD45^+^CD11b^+^ myeloid cells in lung, spleen, liver and heart. (B) Ly6G, Ly6C, Gr1 markers and forward/side scatter parameters were used to define the following myeloid cell subsets: polymorphonuclear myeloid derived cells (PMN-MDSC), monocytic myeloid derived cells (M-MDSC), tumor-associated macrophages (TAM). Analysis was done in 2–3 month-old mice, about one month after *i.p.* injection of tamoxifen. Data are representative of at least three experiments with similar results (error bars, SEM); at least three TG and control animals were analyzed in each experiment. TG/Endo, ROSA26.vFLIP;Cdh5(PAC).creER^T2^; TG/B-cells, ROSA26.vFLIP;CD19.cre mice used as control.

To gain insights into the mechanism and consequences of the cytokine storm and further assess the effect of transgene expression *in vivo*, myeloid differentiation was analyzed by flow cytometry with particular emphasis at the cell subsets that could be influenced by or responsible for the observed cytokine perturbation. A large increase in number of CD45^+^CD11b^+^Gr1^+/−^ cells was found in lung, spleen, liver and heart, both in endothelial and B-cell specific vFLIP TG mice (Fig. 6A). A more detailed analysis revealed that the myeloid subpopulation preferentially expanded was Ly6G^+^Ly6C^int^ (Fig. 6B). These cells were large (FSC^high^), have high granularity (SSC^high^) and expressed high levels of Gr1, therefore they likely represent granulocytic myeloid derived suppressor cells (MDSCs) (also called polymorphonuclear-MDSCs, PMN-MDSCs), as opposed to monocytic-MDSCs (Ly6G^int^Ly6C^+^) that lack granularity and express lower level of Gr1 [41–43]. Moreover, Ly6G^−^Ly6C^−^ cell population, which was expanded only in lung and heart, represented cells that did not express Gr1, were smaller, had no granularity and potentially represent tumor associated macrophages (TAM) or DCs based on immunophenotype, although a functional characterization would be necessary to confirm this. Considering that the endothelial cells are a component of the hematopoietic niches, we compared bone marrow from control and TG mice, but no abnormalities in myeloid or lymphoid hematopoiesis were found that could be ascribed to the expression of vFLIP in endothelial cells.

The tumor microenvironment has been shown to be deeply affected by myeloid cells, including CD11b^+^Gr1^+^ cells, which are able to produce soluble factors, such as Bv8, that influence angiogenesis, extracellular matrix remodeling, anti-VEGF resistance and mobilization of additional myeloid cells toward premetastatic sites [44]. Therefore, we checked whether the expanded myeloid cell subpopulations were differentially expressing any these factors. No differences were observed in TG versus WT mice in the levels of expression of Bv8, VEGF and MMP9, indicating that these cell subsets exert their function in vFLIP-mediated pathogenesis through different mechanisms.

## Discussion

In this study, we have investigated the effect of inducible recombinant vFLIP expression in endothelial cells to model KSHV-associated vascular pathogenesis as observed in KS. Mice developed pathological abnormalities with systemic changes and appearance of elongated spindle-like endothelial cells, mimicking aspects of KS and other KSHV-associated diseases. Mice developed a profound proinflammatory phenotype with perturbation of serum cytokines, similarly to KICS, as well as expansion of myeloid cells, which unveiled a key role of vFLIP in initiating a cascade of events that lead to changes in host microenvironment, ultimately favoring tumor immune evasion, angiogenesis and tumor progression during KSHV pathogenesis.

Given the evidence that KSHV can infect both BECs and LECs [35–37], and vFLIP induces spindling of endothelial cells *in vitro* [27], we tested the hypothesis that *in vivo* expression of vFLIP in endothelial cells would lead to the development of KS-like disease. Mice developed vascular abnormalities with the presence of spindle cells expressing endothelial antigens in virtually all organs, but, unexpectedly, not in the skin, which is the most common location for KS in humans. While the reasons for this finding are unclear, other viral genes are likely to contribute to the many aspects of KSHV pathogenesis in humans in the context of natural infection, including specific organ involvement [45]. Nevertheless, the endothelial-specific vFLIP TG mice generated showed a proliferation of spindle cells, and a proinflammatory phenotype, indicating that this characteristic of KS can be induced by vFLIP alone. In this setting, vFLIP induces expression of cytokines including those that can result in formation of autocrine loops. For example, there is increased production of IL2, and the IL2 receptor alpha chain is upregulated by NF-κB [46], which is turn is activated by vFLIP. Similarly, there is an increase of TNF production in the vFLIP TG mice, and the TNF receptor (CD120B) molecule is induced by NF-κB [47], which in turn can further activate the NF-κB pathway creating a positive regulatory loop. However, we did not obtain complete KS phenotype, so it is likely that cooperation with other KSHV proteins (e.g., vGPCR, LANA, vCyclin, vIL-6, K1) and/or noncoding transcripts (e.g., miR 17–92, miR K12-7), which are co-expressed in KS and relevant for vascular tumorigenesis, are required for full pathogenesis [48–52]. In this regard, previously reported TG mice for vGPCR and vCyclin also failed to fully recapitulate KSHV-associated vascular diseases although tumorigenic properties of the viral products were otherwise demonstrated [48,49,53–58]. Expression of multiple viral products has been achieved in B-cells using the latency locus under the control of the native viral promoter, but specific expression in endothelial cells has not been assessed [24]. Our mouse model contrasts with previous TG models of KSHV-encoded genes in the extent of a proinflammatory phenotype.

The severity and systemic nature of the endothelial changes were reminiscent of certain features of the POEMS syndrome [7]. Although the etiopathogenesis of this syndrome is still largely unknown, a role for KSHV has been suggested by few studies. First, there is frequent association with KSHV-associated MCD and angioma formation. Second, in POEMS syndrome there is overproduction of proinflammatory cytokines, including TNFα, IL1β, IL10, IL6, VEGF [59], similarly to what is observed in MCD and KICS, suggesting that these three clinical entities partially overlap. Moreover, POEMS is characterized by the presence of monoclonal Ig, usually IgG or IgA with lambda light chain, and KSHV encodes for viral IL6 that is functionally active on human myeloma cells [60]. KSHV was found in the lymphoid cells of MCD, as well as in the microvenular hemangioma, the pathognomonic endothelial lesion, positive for CD34, CD31, LYVE-7 and Prox-1, that characterizes this syndrome [61–64]. However, other studies failed KSHV detection in this syndrome [65,66]. Similarities of POEMS with ROSA26.vFLIP;Cdh5(PAC).creER^T2^ TG mice included: i) neuropathic symptoms, which in mice are likely related to hyperplasia of the perineurium around nerve bundles in spinal nerve roots, ganglion and skeletal muscle, ii) systemic presence of elongated endothelial cells, particularly in the heart, which is also increased in size, reminiscent of organomegaly seen in POEMS iii) proneness to develop endocrinopathy (*e.g.*, diabetes), as suggested by increased glycemic levels observed in TG mice, and iv) overproduction of proinflammatory cytokines, including TNFα, IL10, IL6. While the association between POEMS and KSHV remains controversial, a role for KSHV in KICS is well-established and the cytokine storm observed in the vFLIP TG mice is very reminiscent of that seen in this syndrome [4,5].

**Figure 7:**
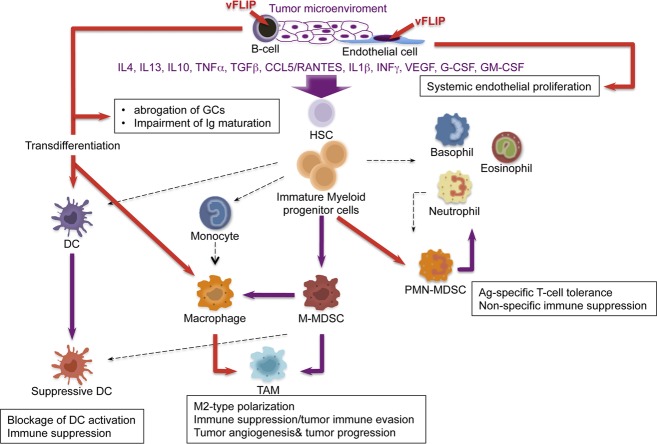
Model of KSHV vFLIP-mediated tumorigenesis through endothelial alterations, aberrant myeloid differentiation, and chronic proinflammatory changes in the tumor microenvironment. Expression of vFLIP in either B-cells or endothelial cells activates several cytokines, both *in vivo* and *in vitro*, that lead to aberrant myeloid differentiation with the emergence of myeloid subsets well known to have a role in angiogenesis, tumor immune evasion and tumor progression. *(Drawing of myeloid differentiation was modified from Gabrilovich et al)[43]*

We also observed remodeling of myeloid differentiation with expansion of CD11b^+^Gr1^+^Ly6G^+^Ly6C^+/−^ cells, phenotypically corresponding to granulocytic myeloid derived suppressor cells (MDSCs). Under physiological conditions, immature myeloid cells from the bone marrow differentiate into granulocytes, macrophages or dendritic cells (Fig. 7). Tumors are capable of secreting several factors in the tumor microenvironment responsible for changes in myeloid differentiation that ultimately can favor tumor immune evasion, angiogenesis and tumor progression. M1 toward M2 polarization is favored by increase in IL10 and reduction in IL12, which lead to reduced Th1 activity and tumor immune evasion, along with angiogenesis and tumor promotion. The main myeloid subpopulations responsible for these effects in tumors are TAM, MDSC, and suppressive DC (Fig. 7). Aberrant CD11b^+^Gr1^+^ myeloid cells have also been found in the mouse placenta, where most likely exert immune suppressive and angiogenetic functions to promote immune tolerance and growth of the developing embryo [67]. Mouse MDSCs consist of two major subsets: granulocytic CD11^+^Ly6G^+^Ly6C^low^ cells and monocytic CD11b^+^Ly6G^+/−^Ly6C^high^ cells (M-MDSCs), which differ in their immunosuppressive mechanisms [43,68]. MDSCs derive from the bone marrow hematopoietic precursors due to the altering of myelopoiesis by chronic inflammatory mediators [69], such as STAT1 and NF-κB, signaling pathways known to be vFLIP targets (56, 59). MDSCs exert their immunosuppressive functions primarily by inhibiting antitumor T-cell function. Moreover, MDSCs are able to secrete angiogenic factors, matrix metalloproteinases and cytokines promoting neoangiogenesis and tumor growth and skewing immune responses towards protumoral Th2-type with activation of Tregs. Thus, MDSCs play a central role in the development of immunosuppressive tumor microenvironment [43], as also emphasized by the fact that functionally active tumor-specific CD8^+^ T-cells can develop anergy or undergo apoptosis when adoptively transferred into a microenvironment containing MDSCs; moreover, depletion of MDSCs restore CD8^+^ T cell function, thus confirming their role in induction and maintenance of host immunosuppression [41].

The cooperation between chronic inflammation and myeloid cell expansion is particularly relevant. In our vFLIP TG mice there is evidence of chronic inflammation at different anatomic sites, sustained also by left-shift in myeloid differentiation. Moreover, vFLIP transcriptome, as defined by *in vitro* gene expression profiling of both vFLIP-expressing endothelial cells and PEL [28,40], highlights the fact that vFLIP activates several proinflammatory cytokines directly implicated in tumor microenvironment and remodeling of myeloid cells, particularly IL4, IL10, IL6, IL13, TGFβ, CCL5/RANTES,IL2, IL1β, G-CSF, similar to those seen in our *in vivo* data. The myeloid phenotype observed in our vFLIP TG mice, with expansion of phenotypically *bona fide* granulocytic-MDSCs, is the first demonstration that vFLIP exerts *in vivo* induction and remodeling of myeloid differentiation with changes in critical components of the microenvironment toward a proinflammatory, angiogenic and immunosuppressive effect. The aberrant myeloid differentiation seems to be a consequence of vFLIP-mediated perturbation of cytokine profiles; once the microenvironment is polarized toward M2, development of MDSCs rather than Th1 activity is favored. In turn, MDSCs, through the upregulation of molecules such as VEGF, Bv8 and MMP9, can favor angiogenesis, tumor progression and tumor immune evasion (Fig. 7). Additional studies are necessary to dissect whether this cytokine storm is produced by myeloid cells or, alternatively, by the endothelial cells with the myeloid cells being a target of this cytokine overproduction. However, the myeloid phenotype with expansion of CD11b^+^Gr1^+^cells was observed in both endothelial and B-cell specific vFLIP TG mice, therefore it likely represents myeloid cells chemotactically recruited by the ectopic expression of vFLIP in either cell type, which, in turn, precedes the expression of cytokines known to have tropism for myeloid cells.

In addition to vFLIP’s ability to impair GC formation and Ig maturation, this change in cytokine profile with remodeling of myeloid differentiation might represent a novel mechanism developed by KSHV to achieve immune evasion by altering the microenvironment to prevent immune recognition of KSHV-infected cells. Considering that Th1-type responses promote cellular immunity against intracellular pathogens and tumors, particularly meaningful is the evidence that KSHV as oncovirus has developed mechanisms to induce Th2 polarization and sabotage host immunity through manipulation of the microenvironment. Interestingly, also KSHV miR-K12-7 induces the expression of IL6 and IL10 [70], which by inhibiting DC maturation protect PEL from host immune recognition [71] and simultaneously act as independent growth factors for these cells [72,73]. It is likely that myeloid differentiation is also perturbed in KSHV-infected individuals, with M2 polarization and impairment of Th1 activity. Although there is need for prospective studies on myeloid cells in KSHV-infected patients, quantitative and functional defects of peripheral blood DC and monocytes with reduced IL12 and increased IL10 were reported as becoming even more pronounced in advanced stages of KS [74]. Moreover, KSHV-specific CTLs are very rare in patients who progress to KS, supporting the role of Th1 immune responses in controlling KSHV replication and transformation [75]. Finally, the cytokine profile from patients with KSHV-associated disease further sustains the hypothesis based on our *in vivo* finding that vFLIP-induced M2 polarization of the microenvironment (with increased IL10, IL13, IL4, INFγ and reduction in IL12) is critical for KSHV pathogenesis.

KSHV is associated with KS in which tumor identity has been made extremely puzzling by the presence of a rich myeloid component, as well as KICS and MCD, both associated with inflammatory cytokines. Our findings suggest this phenomenon is a result of vFLIP-driven remodeling of the microenvironment through a paracrine effect due to the secretion of myeloid-stimulating factors from vFLIP-expressing endothelial or B-cells (Fig. 7). Most macrophages in KS lesions do not contain KSHV, largely favoring a paracrine effect, although rare cells co-express LANA and histiocytic antigens [76].

In conclusion, we have revealed a previously unknown function for vFLIP in inducing *in vivo* expansion of the myeloid compartment with the emergence of a cellular component of immunosuppressive phenotype. This has important implications for the pathogenesis of KSHV-associated malignancies that invariably display a rich myeloid inflammatory infiltrate, which remains poorly characterized. The profound myeloid phenotype induced by vFLIP supports the key role vFLIP has in contributing to host immune dysfunction with development of tumor immune evasion during KSHV pathogenesis. The high-level coordination between cellular and soluble components seen in these mice provide a model to test inhibitors of vFLIP or other immunotherapeutic approaches targeting the microenvironment as potential anticancer agents for KSHV-associated diseases.

## Methods

### Generation of ROSA26.vFLIP;Cdh5(PAC).creER^T2^ TG mouse line

To generate mice expressing the transgene in an endothelial-cell specific manner, homozygous ROSA26.vFLIP TG mice [23] were crossed with heterozygous Cdh5(PAC).creER^T2^ knock-in mice [39] of C57BL/6 genetic background; therefore, all experimental mice were on 129/Sv-C57BL/6 genetic background and age-matched littermates were used as controls. Genotyping was performed by PCR analysis on mouse tail DNA. All mice were housed, bred and studied according to the guidelines of Institutional Animal Care and Use Committee at Cornell University. Mice were monitored for pathological changes weekly and sacrificed when visibly ill, according to approved protocols. Statistical analysis of event-free survival was performed by GraphPad Prism v.5 (San Diego, CA, USA) using Kaplan-Meier cumulative survival curve and the log-rank test to evaluate statistical significance.

### Tissue isolation, reverse-transcriptase polymerase chain reaction (RT-PCR) and immunoblotting

Lung, spleen, liver and heart were isolated during autopsy and promptly processed to obtain a single cell suspension using collagenase A and DNaseI treatment. RNA extraction, RT-PCR and quantitative RT-PCR were performed using standard protocols as detailed in Supporting Methods (S1 Methods). Total protein extracts were prepared from lung, spleen, liver and heart using RIPA buffer, gel electrophoresed on 12% SDS-PAGE gel, transferred to a polyvinylindene difluoride membrane (Millipore) and immunostained according to standard methods using anti-FLAG (M2; Sigma) and anti-β-actin (Sigma) antibodies.

### Flow cytometry

Single-cell suspensions prepared from lung, spleen, liver and heart were stained using standard procedures with a panel of fluorescent-labeled antibodies (see S1 Methods). 7AAD was used for the exclusion of dead cells. Data were acquired on LSRII or Aria flow cytometer (Becton Dickinson) and analyzed using FlowJo software (Tree Star).

### Immunohistochemistry

Four μm thick formalin-fixed, paraffin-embedded sections were stained for H&E or immunostained with the following antibodies: anti-EGFP (Abcam) and anti-CD34 (MEC14.7; Abcam).

### Induction

Mice 8–12 weeks of age were subjected to *i.p.* injection with 0.2 ml of tamoxifen (150 mg) (Sigma), dissolved in a mixture of 90% corn oil (Sigma) and 10% ethanol (Sigma), and analyzed after 30–45 days. Transgene expression was assessed as early as 1 week after induction and remained constitutive over time.

### Cytokine quantification

To determine the concentration of a panel of fourteen serum cytokines, a flow cytometry bead-based assay was used, which exploits particle with discrete fluorescence intensities to detect soluble analytes at very low concentrations. GM-CSF, Phospho Stat1, RANTES, IL12/IL23p40 and MCP1 were quantified using BD Cytometric Bead Array (CBA) Mouse/Rat Soluble Protein Master Buffer Kit, while for the detection of IL10, IL6, INFγ, IL1b, IL12p70, TNF, IL4, IL2, IL13 BD CBA Mouse Enhanced Sensitivity Master Buffer Kit was used. Each capture bead has a distinct fluorescence and is coated with a capture antibody specific for a soluble protein. The bead populations are resolved in two fluorescence channels of a flow cytometry, and each bead population is given an alphanumeric position indicating its position relative to other beads. Beads with different position can be combined to create multiplex assay and analyze multiple proteins from a single sample. After incubation of the capture beads with analytes and detection reagent, the PE mean fluorescence intensity (MFI) of the complex was measured and readings within the assay linear range were used to calculate the serum cytokines concentrations against cytokines standard curve for each analyte (Becton Dickinson).

### Statistical analysis

Statistical significance, defined as *P*<0.05, was assessed by two-tailed unpaired Student’s *t*-test.

## Supporting Information

S1 Methods Materials and methods for flow cytometry, RT-PCR and quantitative real-time RT-PCR. (DOCX)

S1 Fig Endothelial specificity of transgene expression. (DOCX)

S2 Fig Endothelial and astrocytic abnormalities in the brain. (DOCX)

S3 Fig Lack of lymphatic markers in vFLIP-expressing endothelial cells. (DOCX)

S4 Fig Validation of flow cytometry-based assay for quantification of mouse serum cytokines. (DOCX)
