# Correction: Systemic Expression of Kaposi Sarcoma Herpesvirus (KSHV) Vflip in Endothelial Cells Leads to a Profound Proinflammatory Phenotype and Myeloid Lineage Remodeling *In Vivo*

**DOI:** 10.1371/journal.ppat.1013809

**Published:** 2026-01-05

**Authors:** Gianna Ballon, Gunkut Akar, Ethel Cesarman

The Fig 2B TG/B-Cells EGFP panel is incorrect. The updated Fig 2 below presents a corrected EGFP TG/B-Cell panel, along with updated H&E, CD31 and Ki67 panels originating from the same specimen. Furthermore, the original Fig 2 legend included an incorrect reference to PROX-1-stained sections which has been removed from the updated Fig 2 legend below.

Similarities were noted between the Fig 2B TG/Endo EGFP and Ki67 panels and the Fig S3 TG/Endo panel. The corresponding author EC stated that these slides were taken from serial sections of the same muscle specimen and the same areas were selected deliberately for the images shown. Additional panels presenting EGFP and Ki67 stained TG/Endo sections from a second specimen are provided in S1 File.

**Fig 2:**
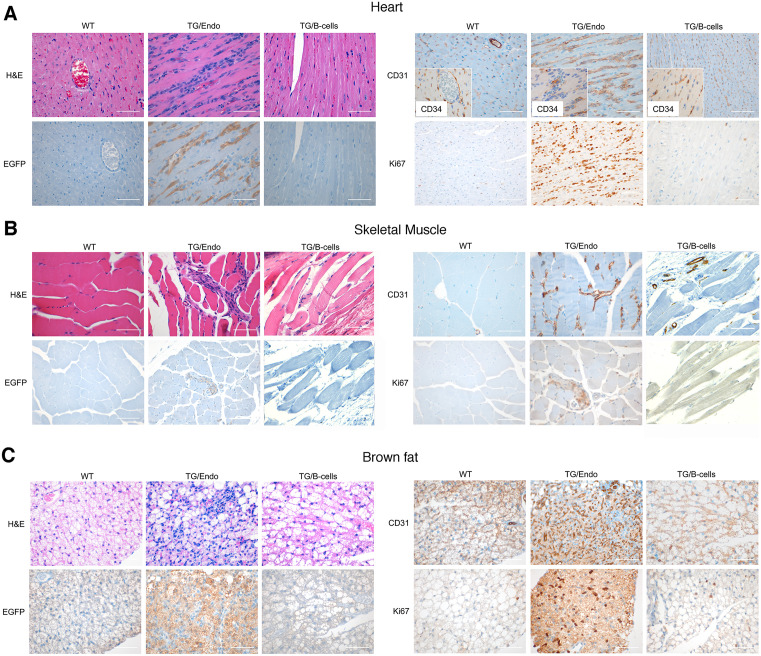
Systemic endothelial abnormalities. **(A)** A representative cardiac section stained with H&E, EGFP and CD34 and/or CD31, and Ki67 shows numerous atypical spindle-like endothelial cells. Similar cells were found in the skeletal muscle (B) and in brown fat **(C)**. Analysis was done in 2–3 month-old mice, about one month after *i.p.* injection of tamoxifen. Scale bar, 200 μm. TG/Endo, ROSA26.vFLIP;Cdh5(PAC).creER^T2^; TG/B-cells, ROSA26.vFLIP;CD19.cre mice used as control.

## Supporting information

S1 File Additional images of EGFP and Ki67 stained skeletal muscle cells from TG/Endo mice, derived from a second specimen and supplementary to their respective panels in Fig 2B. (TIF)
