# Supplementary material for: Systemic Expression of Kaposi Sarcoma Herpesvirus (KSHV) Vflip in Endothelial Cells Leads to a Profound Proinflammatory Phenotype and Myeloid Lineage Remodeling In Vivo
Source: PLoS Pathog. 2015 Jan 21;11(1):e1004581. doi: 10.1371/journal.ppat.1004581 (PMC4301867; doi:10.1371/journal.ppat.1004581)
Supplement: S1 Methods — (DOCX) [file ppat.1004581.s001.docx]

**SUPPORTING METHODS**

**Systemic expression of Kaposi sarcoma herpesvirus (KSHV) vFLIP in endothelial cells leads to a profound proinflammatory phenotype and myeloid lineage remodeling *in vivo***

**Gianna Ballon^1^, Gunkut Akar^1^ and Ethel Cesarman^1^**

^1^Department of Pathology and Laboratory Medicine, Weill Cornell Medical College, 1300 York

Avenue, New York, NY 10065, USA

**Flow Cytometry**

The following fluorescent-labeled anti-mouse antibodies were used: Pacific Blue-conjugated anti-CD19 (6D5; Biologend), PE-conjugated anti-Ly6G and Ly6C (Gr1) (BR6-8C5; BD Pharmingen), PE-conjugated anti-CD31 (PECAM-1) (390; eBioscience), APC-Cy7- or Alexa Fluor700-conjugated anti-CD45 (30-F11; BD Pharmingen), APC-conjugated anti-CD11b (M1/70; BD Pharmingen), Alexa Fluor700-conjugated anti-Ly6G (1A8; BD Pharmingen), V450-conjugated anti-Ly6C (AL-21; BD Pharmingen).

**RT-PCR and quantitative real-time RT-PCR**

Total RNA was isolated from lung, spleen, liver and heart using TRIZOL reagent (Invitrogen), while total RNA from sorted Ly6G^+^Ly6C^int^, Ly6G^int^Ly6C^+^ and Ly6G^-^Ly6C^-^ cells was isolated using Arcturus PicoPure RNA isolation kit (Applied Biosystems). DNase-treated total RNA was reverse transcribed into cDNA using Reverse Transcription System and oligodT primers (Promega), following manufacturer’s instructions, and used for RT-PCR or quantitative RT-PCR. For RT-PCR, two μl of cDNA were added to a PCR reaction containing 10 pmol of each primer, 200 mM dNTP, 10 mM Tris- HCl (pH 8.3), 50 mM KCl, and 4.5 mM MgCl_2_ in a final reaction volume of 25 μl. Initial denaturation was performed at 95°C for 2 min, followed by 34 cycles at 95°C for 15 sec, 56°C for 15 sec, and 72°C for 1 min, followed by a final primer extension at 72°C for 7 min. The following primer sets were used: vFLIP3XFLAG (5’- TTCCACACAGATTCGCACAGA-3’; 5’-GGCACGCCACCAGACAA-3’), CRE (5’- CGTCGGTAGCGGCTTCA-3’; 5’-GGCTATGCCAGCGTCGAGTA-3’), EGFP (5’- GGTGATGTTCTGAGTACATAGCGG-3’; 5’-CCGAGGACGAAATGGAAGTG-3’), β-actin (5’-GGAGTCAACGGATTTGGTCGTA-3’, 5’- GGCAACAATATCCACTTTACCAGAGT-3’). Quantitative RT-PCR on sorted cells was performed using an ABI 7000 Thermal Cycler (Applied Biosystems). Each standard reaction contained 2 μl RT product, 10 pmol of each primer of each primer and SYBR Green PCR master mix (Applied Biosystems) in a final reaction volume of 20 μl, following the manufacturer’s instructions. All samples were tested in triplicates and C_T_ values were calculated. The following primer sets were used: vFLIP (5’- CACTATAGGGTCTCGCAGCA-3’; 5’-GGCGATAGTGTTGGGAGTGT-3’), VEGF (5’-GCTCTACCTCCACCATGCCA-3’; 5’-CACCACTTCGTGATGATTCTG-3’), Bv8 (5’-ACTGCTACTTCTGCTGCTAC-3’; 5’-TGAGACTCGACGGACATTGT-3’), MMP9 (5’-TTGAGTCCGGCAGACAATCC-3’; 5’-CCTTATCCACGCGAATGACG-3’), GAPDH (5’-GTTCCAGTATGACTCCACTC-3’; 5’-CAACCTGGTCCTCAGTGTA-3’), beta-actin (5’- GATGACGATATCGCTGCGCTG-3’; 5’-GTACGACCAGAGGCATACAGG-3’) .

For normalization purposes, ∆C_T_ values were obtained by subtracting GAPDH or beta-actin C_T_ values from those of each tested gene and results are shown as 1/∆C_T_. Negative controls without RNA and without reverse transcriptase were added for each reaction. A representative experiment is shown in which 4 samples per category were analyzed (error bars, SEM).
