# Supplementary material for: Systemic Expression of Kaposi Sarcoma Herpesvirus (KSHV) Vflip in Endothelial Cells Leads to a Profound Proinflammatory Phenotype and Myeloid Lineage Remodeling In Vivo
Source: PLoS Pathog. 2015 Jan 21;11(1):e1004581. doi: 10.1371/journal.ppat.1004581 (PMC4301867; doi:10.1371/journal.ppat.1004581)
Supplement: S1 Fig — (DOCX) [file ppat.1004581.s002.docx]

**
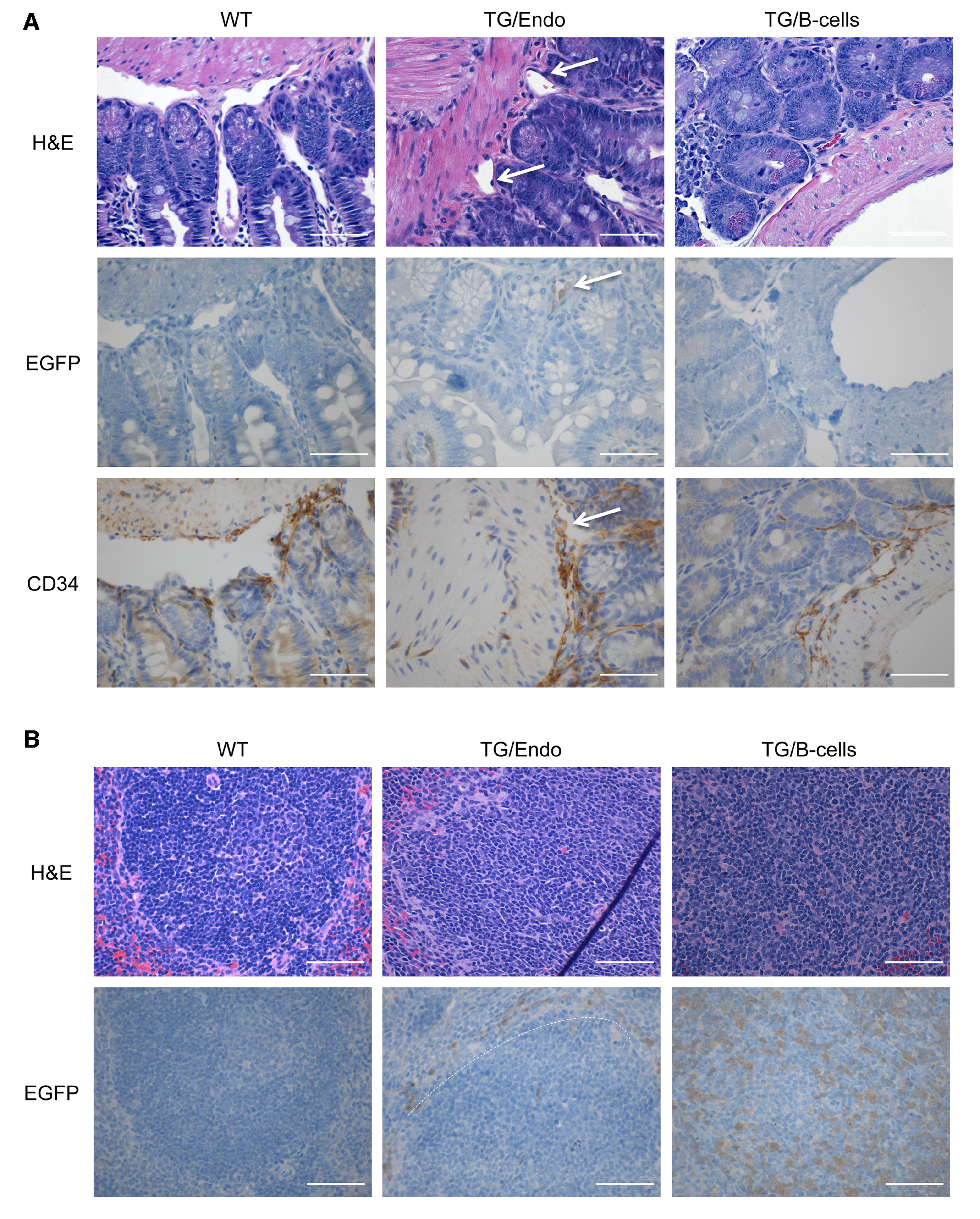
**

**Figure S1. Endothelial specificity of transgene expression.** Histological sections of the intestine (A) and spleen (B) immunostained with H&E, EGFP and CD34 are shown. In the intestine, endothelial cells with fusiforn nuclei and scant cytoplasm are positive for EGFP (arrows). EGFP expression is seen in the vascularized interfollicular area in the spleen (dashed line). Scale bar, 200 μm. TG/Endo, ROSA26.vFLIP;Cdh5(PAC).creER^T2^; TG/B-cells, ROSA26.vFLIP;CD19.cre mice used as control.
