# Supplementary material for: Systemic Expression of Kaposi Sarcoma Herpesvirus (KSHV) Vflip in Endothelial Cells Leads to a Profound Proinflammatory Phenotype and Myeloid Lineage Remodeling In Vivo
Source: PLoS Pathog. 2015 Jan 21;11(1):e1004581. doi: 10.1371/journal.ppat.1004581 (PMC4301867; doi:10.1371/journal.ppat.1004581)
Supplement: S2 Fig — (DOCX) [file ppat.1004581.s003.docx]

**
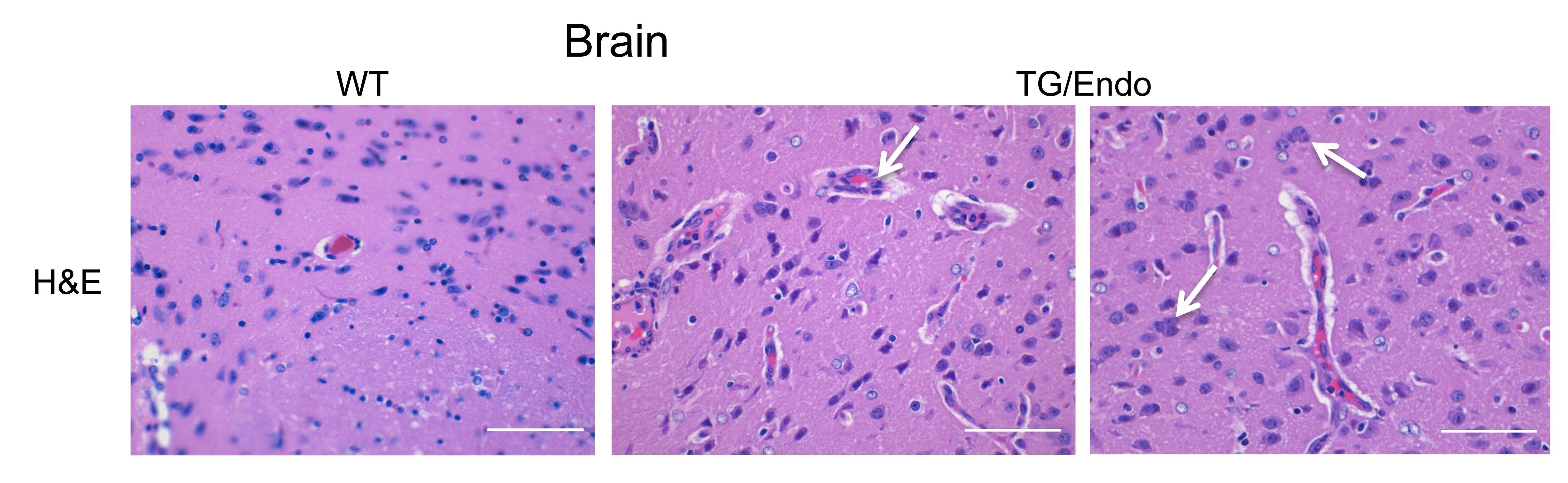
**

**Figure S2. Endothelial and astrocytic abnormalities in the brain.** Midbrain section stained with H&E. Plump endothelial cells (middle panel) and abnormal astrocytes, hypertrophic and with disperse chromatin (right panel) are shown (arrows). Analysis was done in 2-3 month-old mice, about one month after *i.p.* injection of tamoxifen. Scale bar, 200 μm.
