# Supplementary material for: Systemic Expression of Kaposi Sarcoma Herpesvirus (KSHV) Vflip in Endothelial Cells Leads to a Profound Proinflammatory Phenotype and Myeloid Lineage Remodeling In Vivo
Source: PLoS Pathog. 2015 Jan 21;11(1):e1004581. doi: 10.1371/journal.ppat.1004581 (PMC4301867; doi:10.1371/journal.ppat.1004581)
Supplement: S3 Fig — (DOCX) [file ppat.1004581.s004.docx]

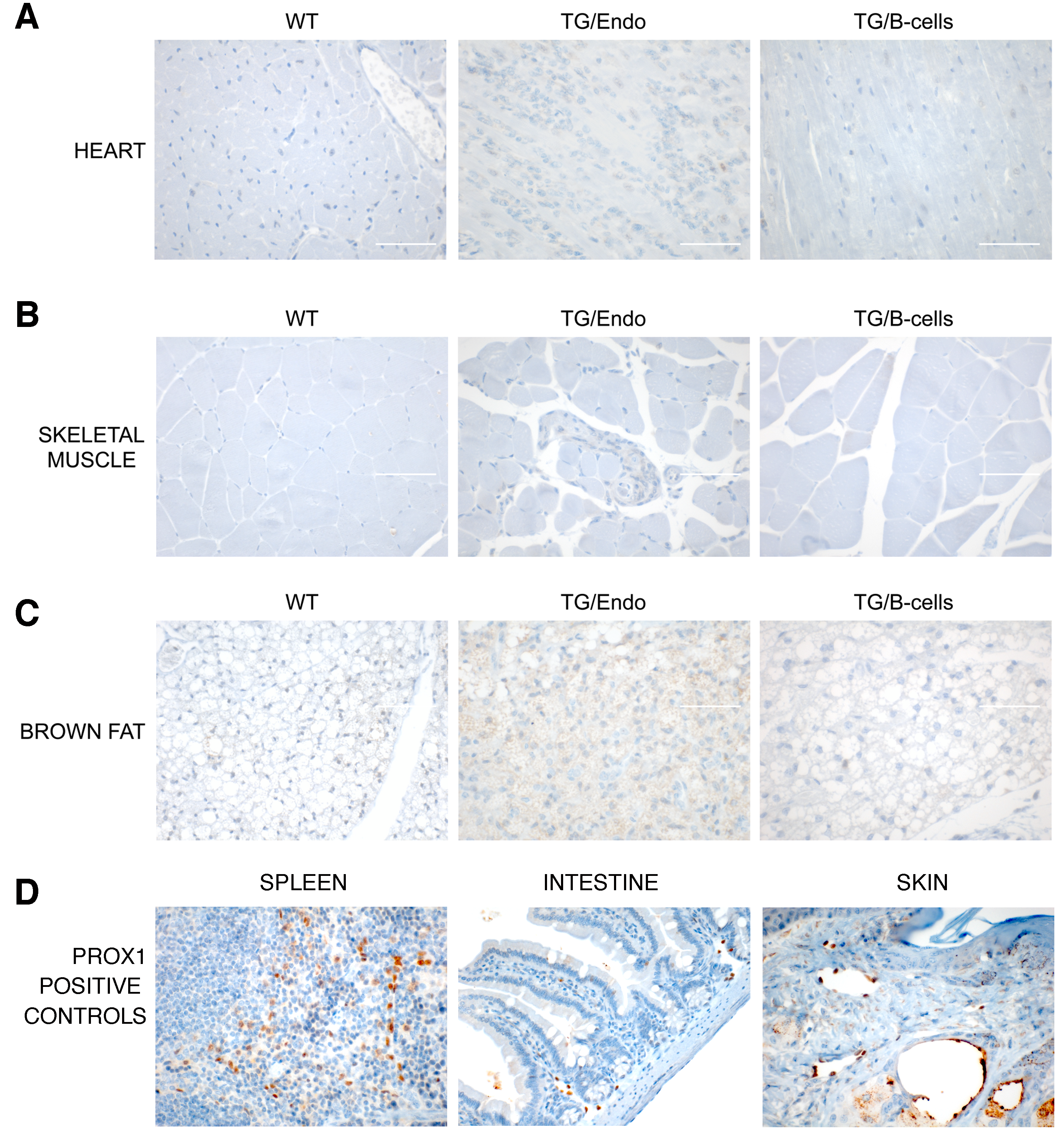


**Figure S3. Lack of lymphatic markers in vFLIP-expressing endothelial cells**. Representative section obtained from the samples were stained with PROX1, showing lack of lymphatic marker expression in heart (A), skeletal muscle (B) and brown fat (C). Positive staining is shown in areas containing lymphatic vessels as indicated (D).
