# Supplementary material for: Systemic Expression of Kaposi Sarcoma Herpesvirus (KSHV) Vflip in Endothelial Cells Leads to a Profound Proinflammatory Phenotype and Myeloid Lineage Remodeling In Vivo
Source: PLoS Pathog. 2015 Jan 21;11(1):e1004581. doi: 10.1371/journal.ppat.1004581 (PMC4301867; doi:10.1371/journal.ppat.1004581)
Supplement: S4 Fig — (DOCX) [file ppat.1004581.s005.docx]

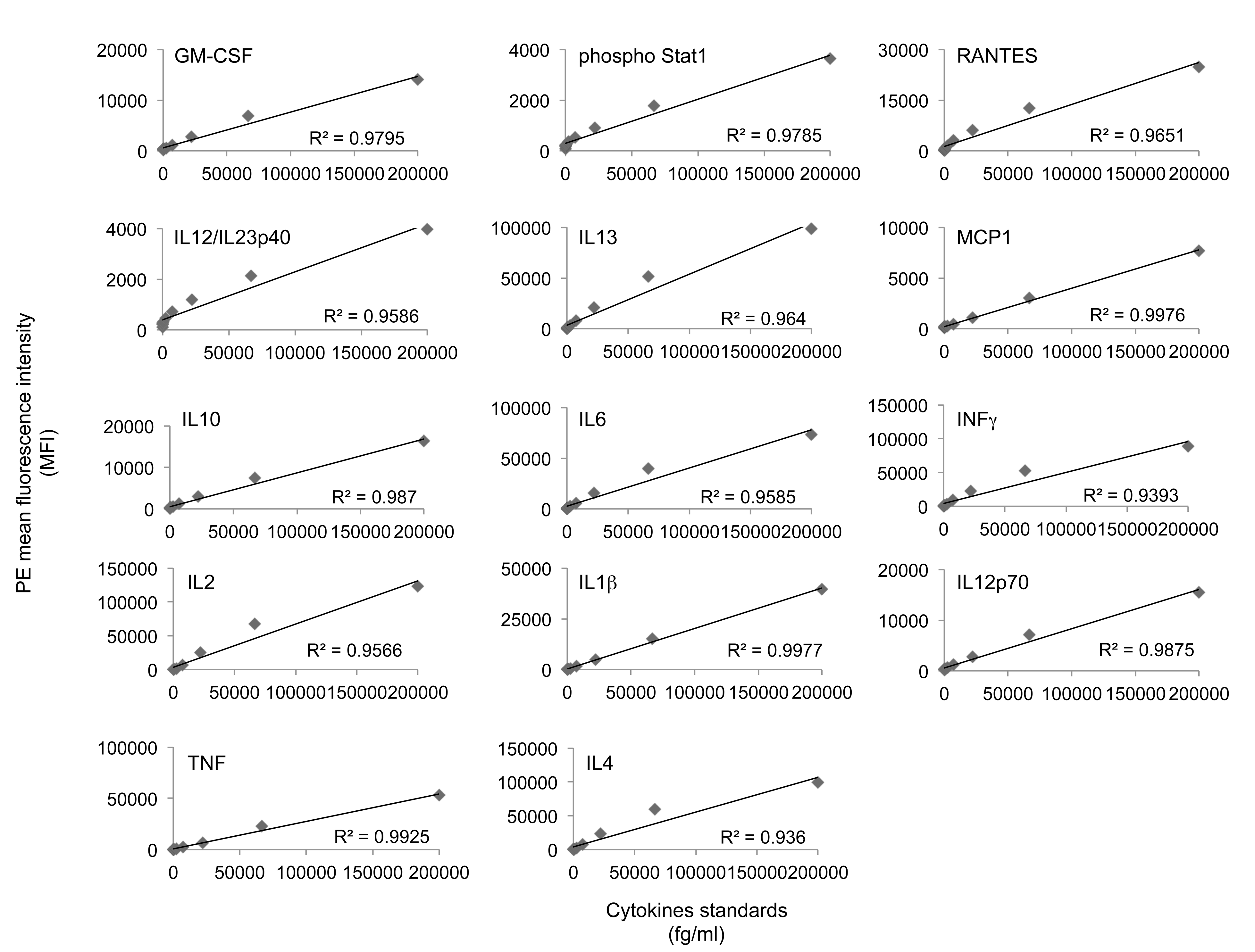


**Figure S4. Validation of flow cytometry-based assay for quantification of mouse serum cytokines**. Cytokines standard curves for each analyte were incubated with capture beads and detection reagent, and the PE mean fluorescence intensity (MFI) of the each complex was measured by flow cytometry. Readings were linear within a large range, from 30 fg/ml to 200000 fg/ml.
