# Supplementary figures and images for: Correction: Systemic Expression of Kaposi Sarcoma Herpesvirus (KSHV) Vflip in Endothelial Cells Leads to a Profound Proinflammatory Phenotype and Myeloid Lineage Remodeling In Vivo
Source: PLoS Pathog. 2026 Jan 5;22(1):e1013809. doi: 10.1371/journal.ppat.1013809 (PMC12768261; doi:10.1371/journal.ppat.1013809)

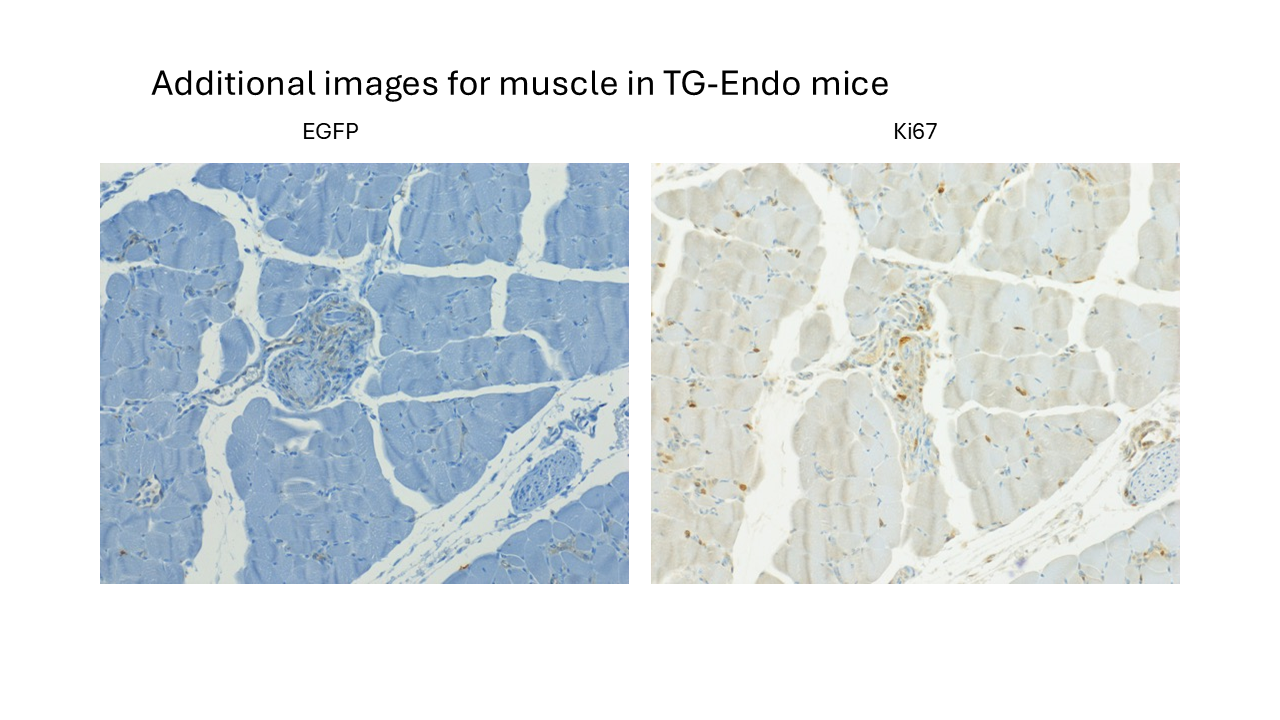

Supplement: S1 File — (TIF) [file ppat.1013809.s001.tif]
